# Is distal segment ostectomy essential for stabilization of the condylar position in patients with facial asymmetry?

**DOI:** 10.1186/s40902-021-00325-3

**Published:** 2021-11-22

**Authors:** Ki Eun Hong, Eun Sup Shin, Jun Park, Ji Eon Yun, Chul Hoon Kim, Jung Han Kim, Bok Joo Kim

**Affiliations:** Department of Oral and Maxillofacial Surgery, College of Medicine, Dong-A University, 26, Daesingongwon-ro, Seo-gu, Busan, 49201 South Korea

**Keywords:** Distal segment ostectomy, BSSRO, Condyle position, Mandibular prognathism

## Abstract

**Background:**

The purpose of this retrospective study was to evaluate the postoperative change in the position and stability of the mandibular condyle after bilateral sagittal split ramus osteotomy (BSSRO) and BSSRO with distal segmental ostectomy (DSO) in patients with facial asymmetry using 3D computed tomography.

**Methods:**

The condyles of the patient diagnosed with facial asymmetry were divided into the deviated side (DS) and the non-deviated side (NDS). Group I, which was treated with BSSRO only, and Group II, which additionally received DSO along with BSSRO, were superimposed on the condyle using the pre-and postoperative 3D CT. The amount of condylar change in anteroposterior displacement, mediolateral displacement, and rotation was measured. The clinical symptoms of temporomandibular joint were also evaluated before and after surgery for each patient.

**Results:**

Between Groups I and II, there was no statistically significant difference in the anteroposterior condylar position on both DS and NDS.

And also, there was no statistical difference between the two groups in the mediolateral change on DS but, statistically significant difference on NDS. The change in the rotation of the condyle was observed to rotate inward from both condylar heads of Groups I and II, and a statistically significant difference was observed between the two groups on both DS and NDS. Moreover, no difference in clinical temporomandibular joint symptoms was observed after surgery in each DS and NDS condyle of the two groups.

**Conclusions:**

As a result of analyzing the condylar position change of the group treated with BSSRO alone and the group treated with BSSRO and DSO in patients with facial asymmetry, there were statistically significant differences in the mediolateral displacement of NDS and the condyle rotation of NDS and DS. However, the anteroposterior condylar position did not show any difference in the bilateral condyles. In addition, since worsening clinical symptoms of bilateral temporomandibular joint were not observed before and after surgery in both groups, it is concluded that it is not necessary to accompany DSO in patients with facial asymmetry (minimum 3 mm, maximum 7 mm).

## Background

Dentofacial deformities can result from a combination of traumatic factors, growth environment, habits that lead to malocclusion, temporal mandibular joint disorders, etc [1].

For correction of dentofacial deformities, surgeons decide on orthognathic surgery often. Orthognathic surgery is a surgical technique to improve facial profile and correct asymmetry and mandibular prognathism. A thorough understanding of dentofacial deformities is essential to successful surgical clinical outcomes.

The most appropriate surgical method is selected to restore masticatory function in patients and improve facial appearance. Le Fort I osteotomy is typically used for the maxilla [2], while sagittal split ramus osteotomy (SSRO), a popular technique introduced by Trauner and Obwegeser [3], is used for the mandible. In some cases, surgery may be accompanied by additional genioplasty.

**Fig. 1:**
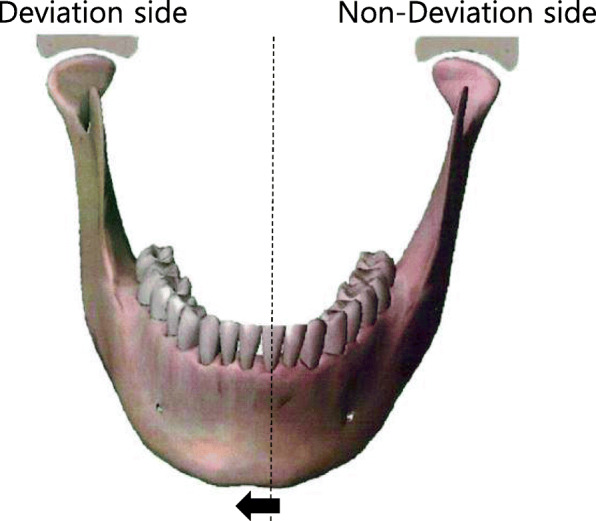
Facial asymmetry. Among the bilateral mandibular condyles, the deviating side of the mandible was assigned as the deviation side and the other condyle as the non-deviation side. The standard of asymmetry is a Menton (Me) deviation (minimum 3 mm, maximum 7 mm)

Recurrence may occur due to incomplete positioning of the mandibular condyle during internal fixation [4]. In addition, in patients with facial asymmetry, distal and proximal bone interference inevitably occurs after BSSRO, which may cause excessive torque on the temporomandibular joint, which may lead to postoperative temporomandibular joint disease. For this reason, clinicians should exercise caution when operating on patients with facial asymmetry (Fig. 1) [5, 6].

Clinically, the incidence of noise, pain, or temporomandibular joint (TMJ) disorders after surgery remains unclear [7] but, the positional change of the mandibular condyle falls within the normal physiological range, in most cases, and problems associated with condylar repositioning are uncommon. However, as mentioned above, patients with facial asymmetry structurally cause a change in the position of the temporomandibular joint more often than patients with only anterior and posterior problems, so additional surgical methods may need to be mobilized. Various mandibular osteotomy has been studied and applied to place the mandibular condyle in the physiological position during the BSSRO procedure [8].

**Fig. 2:**
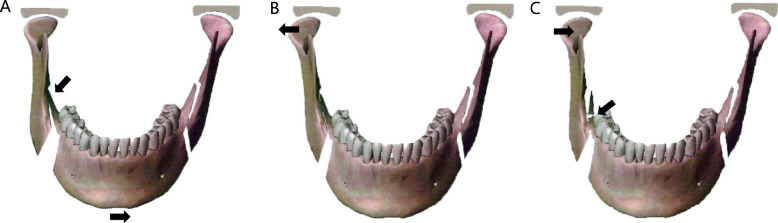
Chin movement after BSSRO. **A** Bony interference occurs between the mesial and distal segment. **B** Torque occurs in the right condyle when the mesial segment is passively aligned. **C** Right condylar reposition by performing DSO

**Fig. 3:**
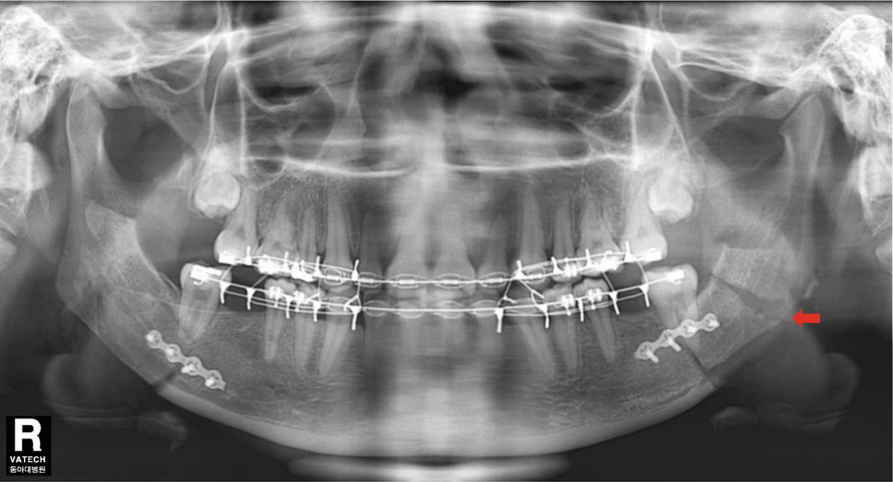
Postoperative panorama of a patient who underwent additional DSO in the left distal segment

DSO is a surgical technique that fractures the posterior molar region of the distal segment and allows for passive contact between the proximal segment and the distal segment. It can eliminate all premature contact areas and prevent nerve compression (Figs. 2 and 3) [9].

The purpose of this retrospective study is to compare and analyze the case of BSSRO alone and BSSRO with DSO in patients with facial asymmetry, confirm the amount of change in the position and clinical symptoms of both condyles, and evaluate the necessity for DSO.

## Materials and methods

From 2016 to 2021, 10 patients who underwent BSSRO and 10 patients who underwent BSSRO and DSO were selected from patients who visited the Department of Oral and Maxillofacial Surgery at Dong-A University Hospital for facial asymmetry with mandibular protrusion. Changes in condylar position and clinical symptoms were evaluated in 20 patients before and 1 month after surgery.

All surgeries were performed by one surgeon. The patients underwent orthodontic treatment before surgery according to their diagnoses.

All patients underwent two-jaw surgery after preoperative evaluation and appropriate treatment plan were established. Lateral cephalometric radiographs, frontal cephalometric radiographs, and 3D-CT were taken for each patient 1 month before and 1 month after surgery. After extracting data from Digital Imaging and Communications in Medicine (DICOM) files of Group I and Group II, the 3D image with the largest diameter of the condylar head in the axial view was selected.

**Fig. 4:**
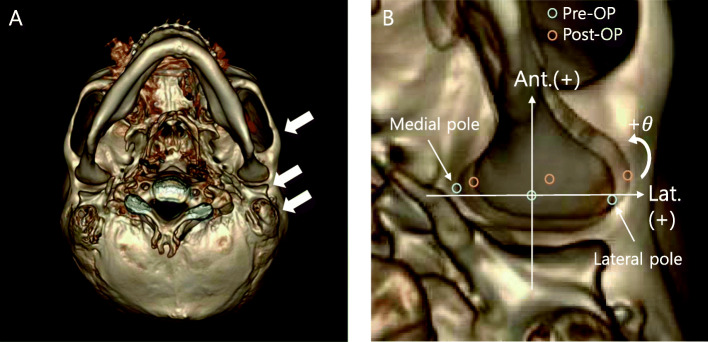
Axial view in 3D-CT: The reference points. **A** The anterior margin of the bilateral zygomatic arch, the posterior glenoid fossa, and the mastoid processes of both temporal bones. **B** Superimposed plane. the midpoint was designated on the axis connecting the medial and lateral poles of the condylar head. In the mediolateral displacement, positive values indicate the lateral direction. In the anteroposterior displacement, the positive values mean the anterior direction. For condyle rotation, the positive values mean inward rotation of condyle head

The pre- and postoperative axial images were superimposed using Photoshop CS3 based on anatomical structures such as the mastoid processes of both temporal bones, the anterior margin of the bilateral zygomatic arch, and the posterior glenoid fossa on both sides (Fig. 4A).

For each patient’s condyles, the chin deviation side was defined as the deviation side (DS) and the other side as the non-deviation side (NDS). In the superimposed images, the midpoint was established on the axis connecting the medial and lateral poles of the condyle head. In Groups I and II, the amount of change in the anteroposterior displacement, mediolateral displacement, and condylar rotation before and after surgery in both condyles was measured based on the midpoint.

In the anteroposterior displacement, the positive values mean the anterior direction. In the mediolateral displacement, the positive values indicate the lateral direction. For condyle rotation, the amount of angular change was measured using the axis connecting the medial pole and the lateral pole. The positive values mean inward rotation of condyle head (Fig. 4B).

The presence or absence of three TMJ symptoms for pain, noise, and LOM was evaluated and compared in both groups before and after surgery. The medical records were used to compare the symptoms before and about 3 months after surgery, and 1 point was assigned to each symptom to calculate the score. The scores were summed for each patient between groups and compared.

### Inclusion and exclusion criteria

Patients who met the following criteria were included: (a) age over 18 years with complete skeletal growth; (b) facial asymmetry and prognathism; (c) chin deviation of 3 mm to 7 mm; (d) a history of Le Fort I osteotomy and BSSRO surgery or additional DSO; and (e) 3D CT scans obtained 1 month before and after surgery.

Exclusion criteria were as follows: (a) history of BSSRO alone without Le Fort I osteotomy; (b) history of surgery for mandibular retrognathism; and (c) presence of TMJ deformity.

### Statistical analysis

The data are presented as frequency and percentage for categorical variables and mean ± standard deviation (SD)/median (interquartile range (IQR)) for numeric variables. Differences in participant characteristics were compared across subgroups using an independent *t* test or the Mann-Whitney *U* test for continuous variables.

Differences in participant characteristics were compared across subgroups using analysis of variance (ANOVA) via Scheffe’s post hoc test or the Kruskal-Wallis test with Dunn’s post hoc test. To test for normal distribution, we used the Shapiro-Wilk test. For graphical visualization, scatter plots with error bar charts were used.

All statistical analyses were performed using SPSS 26.0, and *p* values less than 0.05 were considered statistically significant.

## Results

The average age was 20 years for Group I patients and 22 years for Group II patients. Preoperative chin deviation was observed in patients with a minimum of 3 mm and a maximum of 7 mm, and an asymmetry of 4 mm was observed on average. In patients with a chin deviation of 3 mm or less, there was no significant change in the position of the mandibular condyle, so there was little need for additional DSO or IVRO. For patients with a chin deviation of 7 mm or more, it was judged that it would be difficult to position the mandibular condylar position within the physiological range with DSO alone, and IVRO was often performed.

All 20 patients were accompanied by LeFort I surgery, and mandibular BSSRO alone or additional DSO was performed. Group I consisted of 10 patients who underwent BSSRO alone, and Group II consisted of 10 patients who underwent both BSSRO and DSO. Table 1 shows the amount of chin deviation of the patient before surgery.

**Table 1:** Each patient’s chin deviation. Group I and Group II

Variable	Chin deviation
Group I	
P1	3
P2	3
P3	3
P4	3
P5	4
P6	4
P7	4
P8	5
P9	5
P10	7
Group II	
P1	3
P2	3
P3	3
P4	3
P5	3
P6	4
P7	4
P8	5
P9	5
P10	7

**Fig. 5:**
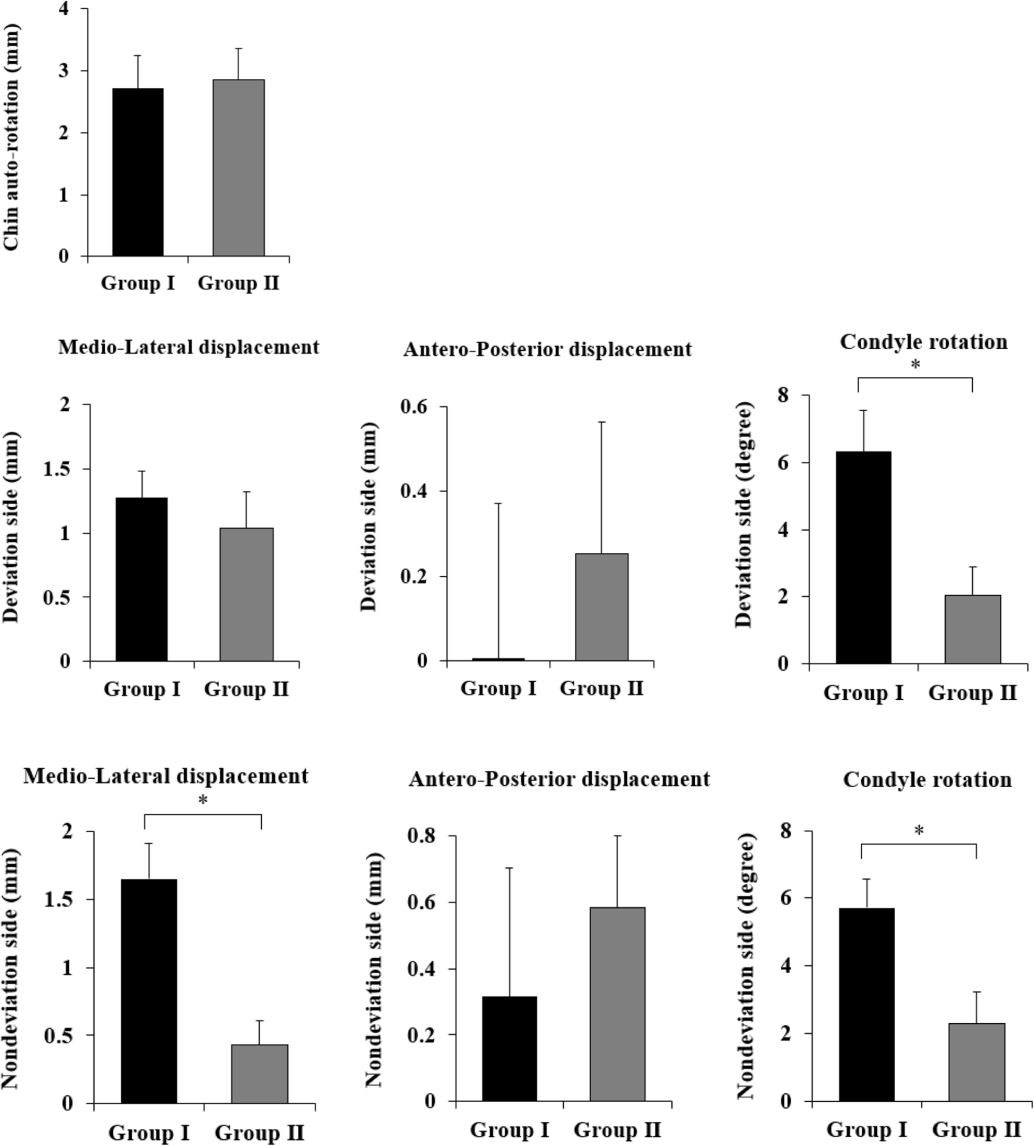
Comparison of anteroposterior displacement, mediolateral displacement, condylar rotation in Group I and Group II on the deviation side and non-deviation side

Table 2 and Fig. 5 show the amount of change in the anteroposterior displacement, mediolateral displacement, and condylar rotation for DS and NDS in Groups I and II, respectively.

**Table 2:** Change in position of mandibular condyle of Group I and Group II

Variable	Group I	Group II	***p*** value
**Medio-lateral displacement**			
Deviation side (mm)			
Mean ± SD	1.27±0.66	1.04±0.89	.510^a^
Median (IQR)	1.46 (0.50–1.84)	1.28 (0.11–1.89)	
Non-deviation side (mm)			
Mean ± SD	1.65±0.83	0.43±0.56	.002^b^
Median (IQR)	1.91 (0.95–2.27)	0.16 (0.09–0.82)	
**Antero-posterior displacement**			
Deviation side (mm)			
Mean ± SD	0.01±1.15	0.25±0.98	.613^a^
Median (IQR)	− 0.39 (− 0.87–0.66)	0.32 (− 0.78–0.81)	
Non-deviation side (mm)			
Mean ± SD	0.32±1.22	0.58±0.68	.552^a^
Median (IQR)	0.30 (− 0.86–1.20)	0.43 (0.03–1.07)	
**Condyle rotation**			
Deviation side (mm)			
Mean ± SD	6.33±3.83	2.05±2.61	.009^a^
Median (IQR)	6.75 (2.88–9.90)	2.50 (0.50–4.25)	
Non-deviation side (mm)			
Mean ± SD	5.71±2.73	2.30±2.91	.015^a^
Median (IQR)	5.05 (3.58–7.48)	2.50 (1.00–4.50)	

Data are presented as mean ± SD and median (IQR)

^a^*P* values were derived from independent *t* test

^b^*P* values were derived from Mann-Whitney’s *U* test

Shapiro-Wilk’s test was employed for test of normality assumption

The amount of change in the mediolateral displacement was 1.27±0.66 mm DS in Group I and 1.04±0.89 mm in Group II (*p* = .510), with no statistically significant difference. However, on NDS, there was a statistically significant difference in the amount of change in Group I (1.65±0.83 mm) and Group II (0.43±0.56 mm) (*p* = .002).

The change in the anteroposterior displacement was 0.01±1.15 mm in Group I DS, 0.25±0.98 mm in Group II (*p* = .613), 0.32±1.22 mm in Group I NDS, and 0.58±0.68 mm in Group II (*p* = .552). There was no statistically significant difference between the two groups in DS and NDS.

The condyle rotation variation was 6.33±3.83° in the deviation side of Group I and 2.05±2.61° in Group II (*p* = .009), with statistically significant difference between the two groups. For NDS, we observed a change of 5.71±2.73° in Group I and 2.30±2.91° in Group II (*p* = .015). There was a statistically significant difference.

In Group I, 6 out of 10 patients had TMJ symptoms before surgery. A decrease in symptoms was observed in 5 out of 6 patients. In Group II, 5 out of 10 patients had TMJ symptoms, and 4 out of 5 patients had symptomatic reduction after surgery (Table 3).

**Table 3:** TMJ Clinical Symptoms Score. If a patient had symptoms corresponding to TMJ-related pain, noise, or limitation of mouth opening before and after surgery, 1 point was assigned to each symptom to calculate a score. Group I and Group II

Variable	Score	
	Pre-OP	Post-OP
Group I		
P1	2	1
P2	0	0
P3	1	1
P4	0	0
P5	0	0
P6	1	0
P7	2	1
P8	1	0
P9	1	0
P10	0	0
Group II		
P1	2	0
P2	1	0
P3	0	0
P4	0	0
P5	1	1
P6	1	0
P7	0	0
P8	0	0
P9	0	0
P10	1	0

## Discussion

In the past literature, superimposition of cephalometric radiography was often used to observe condyle changes after orthognathic surgery. However, it is now possible to observe changes in the position, angle, and shape of the condyle using 3D images in CT [10]. There are two methods for superimposing images: an anatomical structure-based approach and a voxel-based approach [11]. In this study, the changes in the position and angle of the condyle were observed by superimposing based on the anatomical structures.

Many studies have reported that the position of the mandibular condyle changes after orthognathic surgery. Choi et al. [12] reported that the condylar head of patients who underwent orthognathic surgery tended to rotate inward in the axial view and return to its original position 6 months after surgery. Lee et al. [13] reported that the condylar head rotated at an average of 4.00° in the axial plane after BSSRO. The authors also reported that condylar position changes greater than an average of 4° require caution as they may reduce surgical stability or lead to iatrogenic TMJ disorders. Edward Ellis III et al. [14] reported that during orthognathic surgery in patients with severe facial asymmetry, the position of the mandibular condyle is changed due to bone interference between the mesial and distal regions, which may cause TMJ problems. Therefore, he said it was important to properly position the mandibular condyle in the mandibular fossa during the surgical procedure.

For this reason, various studies on bone fixation methods using wires, devices, and plates have been reported to position the condyle within the physiological range during orthognathic surgery [11, 15, 16]. In addition, various modified osteotomy methods have been studied to remove bony interference [17]. Yang et al. [18] evaluated the usefulness of grinding technique and posterior bending osteotomy to prevent bony interference during orthognathic surgery in patients with facial asymmetry of 4 mm or more. According to the study, in the presence of large bony interference, there was a statistically significant tendency for the condyle to rotate inward in the group using the grinding technique. Kim et al. [19] recommended the use of the DSO procedure to minimize the recurrence of patients with facial asymmetry. They reported that, as a result of long-term follow-up, the postoperative recurrence rate was lower in the group with BSSRO and DSO than in the group treated with BSSRO alone, and more stable surgical results were obtained. Based on these studies, in this study, DSO, which can easily remove bone interference, was additionally selected and studied in patients with mild facial asymmetry among patients who planned BSSRO surgery, which has a wider contact area between segments than IVRO.

Although DSO is a useful procedure, care must be taken to prevent nerve damage during DSO because the inferior alveolar nerve is close and it is not routinely performed during orthognathic surgery. However, the risk of nerve damage associated with DSO has not yet been precisely identified [14].

We hypothesized that the change in displacement from the deviation side to the lateral direction would be minimized in Group II, but there was no statistically significant difference between the two groups. In general, during BSSRO surgery, the surgeon should strive to remove most of the interference site between the proximal and distal bone segments to allow for passive contact. Although it is a time-consuming step, there is no doubt that it must be considered, especially in mandibular surgery for patients with facial asymmetry, since reducing bone interference minimizes the mandibular condylar torque. In this study, when interference was removed using DSO, there was no significant difference in displacement between the deviation aspects of Group I and Group II. Therefore, it was assumed that it is not necessary to perform BSSRO along with DSO for patients with mild facial asymmetry with an average asymmetry of about 4 mm (minimum 3 mm, maximum 7 mm) even for patients with facial asymmetry. However, it is considered that additional research is needed on cases where facial asymmetry is excessive by more than 7 mm.

The tendency of inward rotation of the mandibular condyle in DS and NDS was clearly confirmed in all patients as in other studies. This means that when the mandible is repositioned to its normal position, some interference is inevitably generated below the proximal segment including the condyle. However, since it does not affect the manifestation of clinical symptoms, rotation of the mandibular condyle by 5–6 degrees is considered to be within the physiological range.

According to the results of this study, DSO is not an essential procedure in patients with mild facial asymmetry. But attention must be paid to nerve damage when BSSRO is performed on a patient with excessive facial asymmetry of 7 mm or more, which is expected to have severe bone fragment interference. One option for preventing nerve damage is using intraoral vertical ramus osteotomy (IVRO) procedure. IVRO relieves symptoms in patients with TMJ disorders by restoring the TMJ and disc relationship [20]. Furthermore, the procedure is simpler than SSRO, with a shorter operation time and lower risk of nerve damage. However, there are complications such as condylar displacement or luxation [21, 22].

In order to evaluate the stabilization of the condylar position according to each surgical method, a long-term study on the position change of the mandibular condyle, symptoms, and disorders of TMJ after surgery for IVRO and BSSRO with DSO is additionally needed.

## Conclusion

For patients with mild facial asymmetry (avg. 4 mm), additional DSO is not essential to minimize changes in the position of the mandibular condyle and prevent TMJ symptoms during BSSRO surgery.

## Data Availability

The datasets used and/or analyzed during the current study are available from the corresponding author on reasonable request.

## Abbreviations

BSSRO: Bilateral sagittal split ramus osteotomy
DSO: Distal segment ostectomy
IVRO: Intraoral vertical ramus osteotomy
SSRO: Sagittal split ramus osteotomy
3D-CT: Three-dimensional computed tomography
TMJ: Temporomandibular joint
